# Survival and Complications in Pediatric Patients With Cancer and COVID-19: A Meta-Analysis

**DOI:** 10.3389/fonc.2020.608282

**Published:** 2021-01-21

**Authors:** Elisa Dorantes-Acosta, Diana Ávila-Montiel, Miguel Klünder-Klünder, Luis Juárez-Villegas, Horacio Márquez-González

**Affiliations:** ^1^ Biobanco de Investigación en Células Leucémicas, Hospital Infantil de México Federico Gómez, México City, Mexico; ^2^ Subdirección de Investigación, Hospital Infantil de México Federico Gómez, México City, Mexico; ^3^ Departamento de Onco-Hematología, Hospital Infantil de México Federico Gómez, México City, Mexico; ^4^ Investigación Clínica, Hospital Infantil de México Federico Gómez, México City, Mexico; ^5^ Cardiopatías Congénitas. Hospital de Cardiología, Centro Médico Nacional Siglo XXI, Instituto Mexicano del Seguro Social (IMSS), México City, Mexico

**Keywords:** severe acute respiratory syndrome coronavirus 2, coronavirus disease 2019, childhood cancer, meta-analysis, systematic review, mortality, ventilation, intensive care

## Abstract

**Background:**

The pandemic caused by the novel severe acute respiratory syndrome coronavirus 2 (SARS-CoV-2) has affected all age groups, including the pediatric population, in 3–5% of all cases. We performed a meta-analysis to understand the survival and associated complications in pediatric cancer patients as well as their hospitalization, intensive care, and ventilation care (supplemental oxygen/endotracheal intubation) needs.

**Methods:**

A systematic search was performed using MEDLINE, TRIP Database, International Clinical Trials Registry Platform (WHO), The Cochrane Library, Wiley, LILACS, and Google Scholar. Additionally, a search using the snowball method was performed in *Nature*, *New England Journal of Medicine*, *Science*, *JAMA*, *ELSEVIER editorial*, *Oxford University Press*, *The Lancet*, and *MedRxiv*. Searches were conducted until July 18, 2020. A total of 191 cancer patients with coronavirus disease 2019 (COVID-19) were integrated from 15 eligible studies. In a sub-analysis, patients were stratified into two groups: hematological cancer and solid tumors. Outcome measures were overall survival, risk of hospitalized or needing intensive care, and need for ventilatory support in any modality. The random effects statistical analysis was performed with Cochran’s chi square test. The odds ratio (OR) and heterogeneity were calculated using the I^2^ test.

**Results:**

The overall survival was 99.4%. There were no statistically significant differences in the risk of hospitalization between hematological malignancies and solid tumors (95% confidence interval [CI] 0.48–18.3; OR = 2.94). The risk of being admitted to the intensive care unit was also not different between hematological malignancies and other tumors (95% CI 0.35–5.81; OR = 1.42). No differences were found for the need of ventilatory support (95% CI 0.14–3.35; OR = 0.68). Although all the studies were cross-sectional, the mortality of these patients was 0.6% at the time of analysis.

**Conclusions:**

In the analyzed literature, survival in the studied group of patients with COVID-19 was very high. Suffering from hematological neoplasia or other solid tumors and COVID-19 was not a risk factor in children with cancer for the analyzed outcomes.

## Introduction

Up to September 1, 2020, infection by the novel severe acute respiratory syndrome coronavirus 2 (SARS-CoV-2) causing coronavirus disease 2019 (COVID-19) that emerged in China, has caused 25,559,850 infections and 852,109 deaths. Although the exact number of infected children aged <18 years is imprecise, it is estimated to represent 1–5% of the total infections. Therefore, it is likely that >1,000,000 children are infected with this virus (1).

The severity and fatal outcomes of patients infected by SARS-CoV-2 are directly related to age, as demonstrated by Zhang et al., with a higher frequency of hospitalizations, mechanical ventilation requirement, and mortality in individuals aged >60 years (2). Simultaneously, pediatric patients have a limited capacity to follow basic measures to prevent contagion, thus creating a situation of public health risk (3).

The reported SARS-CoV-2 infection presents differences between adults and children, constituting a less severe and even asymptomatic condition in the latter (2). In China, Dong et al. published the first pediatric series in 2,143 children with a median age of 7 years and suspected SARS-CoV-2 infection, of which 728 (34.1%) were confirmed using polymerase chain reaction (PCR) tests, and one adolescent male died (4). Similar outcomes were found in New York, which was one of the main epidemic centers. In this city, Richardson et al. described the characteristics of 5,700 infected patients, of which systemic arterial hypertension (SAH) represented 55.6% of the comorbidities; 34 (6%) of patients were aged <18 years, and no deaths were reported (5).

For the infected pediatric population, Pathak et al. conducted a multicenter study in pediatric intensive care units that included 74 children and that was used as a base for a projection of 50,000 children with severe SARS-CoV-2 disease, of which 5,400 might need mechanical ventilation (6).

Considering that COVID-19 in adults has shown an association between the most prevalent comorbidities (obesity, SAH, diabetes) and the development of severe respiratory complications, it is pertinent to perform the same analysis in the most frequent pediatric diseases, including cancer, one of the main causes of death in the world (7). In this regard, Chen et al. documented the first case in a child with acute lymphoblastic leukemia under treatment and infected with SARS-CoV-2 that simultaneously manifested with a neutropenic event and fever, for which the dynamic outcome was not clarified (8). A series of cases have been published from other pediatric cancer centers in countries that have been epicenters of the COVID-19 pandemic.

Because children with cancer require continuous chemotherapy, the dilemma arises whether to interrupt the therapy or to add up a risk condition that exposes them to the complications described so far caused by infection with SARS-CoV-2. The accelerated spread of the virus has led some international cooperative cancer centers to issue recommendations based on expert consensus (3, 9).

Based on the scientific evidence published to date, it is necessary to assess whether COVID-19 increases the risk of severe outcomes in patients with pediatric cancer.

### Objectives

- To determine the difference in mortality in pediatric patients with cancer and COVID-19 with hematological cancer *vs*. solid tumors.
- To determine the risk of requiring hospitalization, intensive care, and ventilation in patients with COVID-19 and hematological cancer *vs.* solid tumors.

## Materials and Methods

### Type of Studies

Owing to the fact that brief reports were published as letters to the editor in the initial months, articles with the following characteristics were included: cross-sectional or longitudinal observational studies (included in opinion sections or letters to the editor) of series of children with cancer.

Articles that described a single patient and those publications that did not indicate at least one outcome were excluded.

### Type of Participants 

Patients aged <18 years with a diagnosis of cancer and a positive test for SARS-CoV-2 (by PCR) with reference to the outcomes of interest were included. Patients who underwent a transplant or patients with cancer surveillance for >5 years were excluded.

### Variables

Studies that used the PCR test to diagnose the disease were included. The exposure variable was the type of cancer, which was grouped as hematological cancer (acute leukemias and lymphomas) and solid tumors (central nervous system tumors, germ cell tumors, hepatoblastoma, neuroblastoma, osteosarcoma, retinoblastoma, sarcomas, rhabdomyosarcoma, and Wilms’ tumor).

### Outcomes

The outcomes recorded were mortality, need for hospitalization, intensive care unit (ICU) care, and ventilation requirement (including invasive and non-invasive ventilation modalities).

### Search Strategy 

An exploratory systematic literature review was conducted for the period December 2019 to July 18, 2020 to compare the overall and stratified survival in hematological neoplasms and solid tumors in children aged <18 years having cancer and COVID-19.

**Graph 1:**
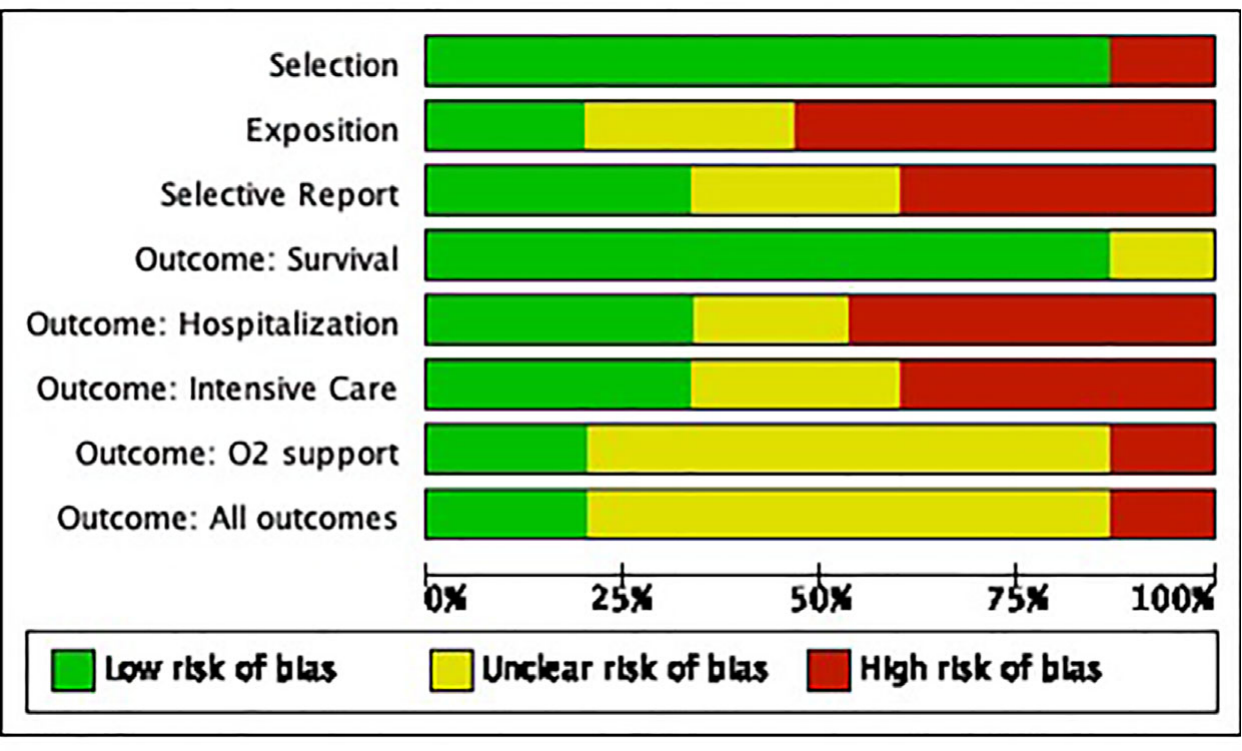
Risk of bias graph of included studies of SARS CoV-2 and cancer in the pediatric population.

The information sources used were MEDLINE, TRIP Database, International Clinical Trials Registry Platform (WHO), The Cochrane Library, Wiley, LILACS, and Google Scholar. (**Graph 1**).

Additionally, the following journals were searched (snowball method): Nature, New England Journal of Medicine, Science, JAMA, editorial de ELSEVIER, Oxford University Press, The Lancet, and MedRxiv. MEDLINE searches were conducted using MESH terms and keywords, without methodological filters by type of article, but only article in English idiom were included.

Additionally, publications in the reference list of the retrieved full text articles were searched to identify additional relevant studies (snowball method).

The search terms used were keywords or MESH terms (for the pediatric stage, the following were used: *pediatrics, children, child, infancy, infant, scholar*, and *adolescent*; for cancer, the following were used: *oncology, hematoncologic, cancer*, and *solid tumors*; for the COVID-19 disease, the following were used: *SARS-COV-2, COVID-19*, and *coronavirus 19*; and for death, the following were used: *mortality, survival*, and *death*).

### Data Extraction

Two reviewers independently assessed the eligibility of the studies for inclusion; the relevant ones were retrieved (according to the eligibility criteria) and subsequently, the necessary information on the characteristics of the included studies was extracted, including participant characteristics, type of exposure, and outcome variables. In both phases, the two reviewers resolved disagreements by consensus; if a disagreement persisted, a third reviewer was consulted. The data obtained were integrated into evidence tables and were verified by the two reviewers. The following information was collected: country of origin, type of design, study objective, study results, if the patient was admitted to the hospital or required ventilation or intensive care, and clinical outcome.

### Bias Control

Bias was controlled using the ROBINS-1 tool for observational studies considering four types of biases: comparability, selection, selective reporting, and outcomes, stratified as present, absent, or doubtful. They were adjusted for each outcome variable (10).

### Statistical Analysis 

Quantitative synthesis was performed by meta-analysis using the Cochrane Review software RevMan 5.3. The analysis was performed with the random effect method, and the risk was calculated with odds ratio (OR) and confidence intervals (95% CI); statistical significance was determined with the Cochran’s chi square test. The heterogeneity of the studies was calculated with the Tau and I^2^ tests. A p value of <0.05 was considered statistically significant.

## Results

**Figure 1:**
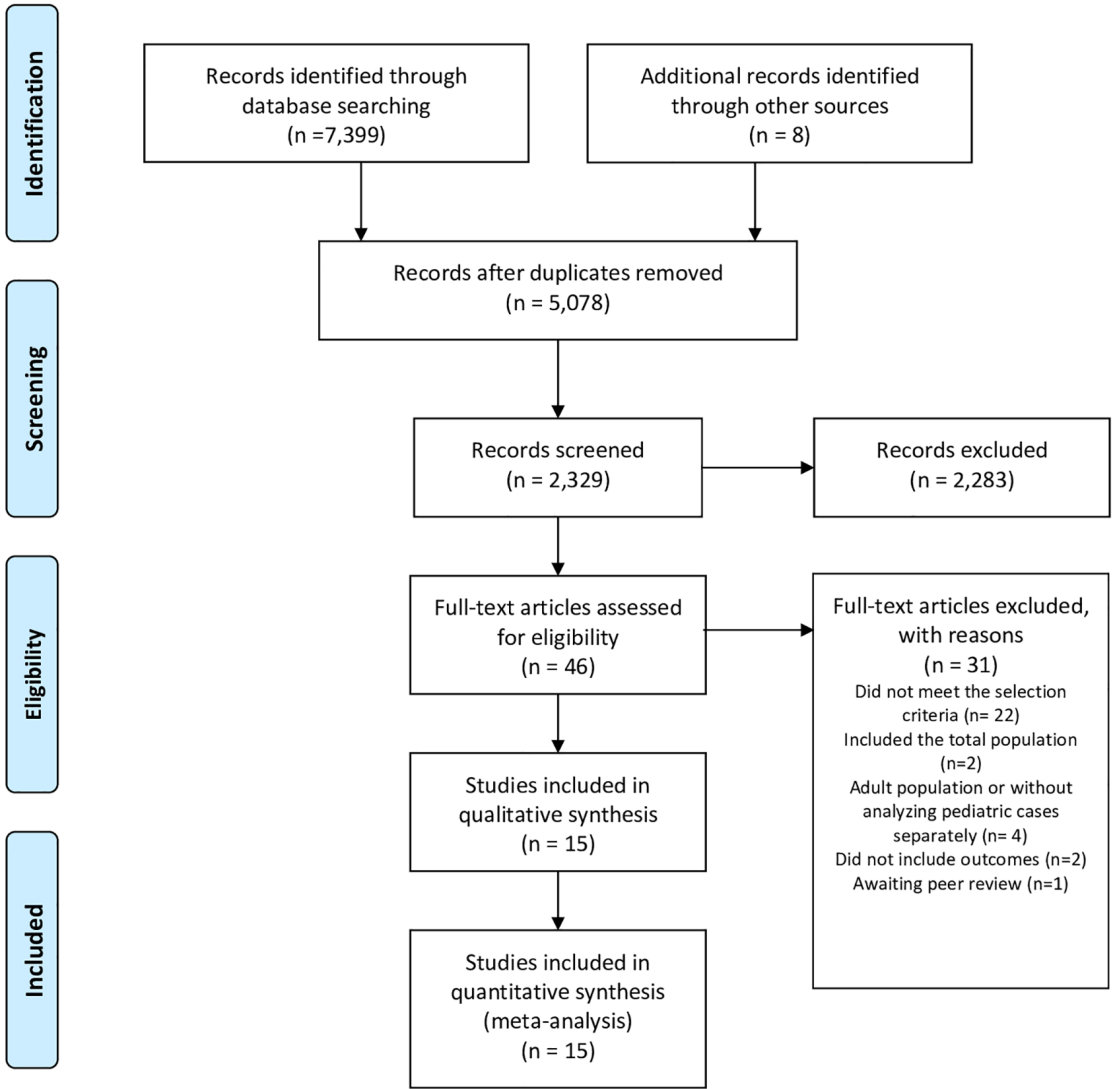
Flow Diagram of included studies.

In the consulted databases, 7,399 articles were identified and eight more from other sources (gray literature), of which 5,078 manuscripts did not match the topic or meet the selection criteria or were repeated. Out of 2,329 abstracts, 46 documents were selected and read in their entirety (**Figure 1**). Finally, 31 articles were excluded for the following reasons: 22 articles did not meet the selection criteria (4, 11–30); four articles included the adult population, and it was not possible to identify pediatric cases (2, 31, 32); two articles did not include outcomes (33, 34) in these cases, the authors were contacted, and in the absence of a response, the articles were excluded—and one article was awaiting peer review (35).

### Articles Included

**Graph 2:**
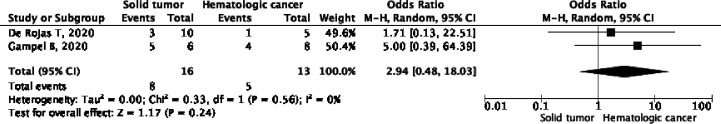
Differences in the need for hospitalization of hematological cancer and solid tumors among patients with SARS CoV-2.

We included 15 articles (3, 36–49) enclosed you will find the **Graph 2** with the risk of bias summary (see **Graph 1**).

### Mortality and COVID-19 in Children With Cancer

Fifteen studies showed survival data from 191 patients with cancer and COVID-19 (3, 45) and recorded the death of a patient with Burkitt lymphoma, a type of non-Hodgkin lymphoma. The studies showed an overall mortality of 0.6% (**Supplementary Table 1**).

### Requirement for Hospitalization

Two studies (40, 43) [de Rojas et al. (40) and Gampel et al. (43)] enabled the analysis of required hospitalization comparing patients with hematological cancer and those with solid tumors. These studies reported the information from 29 patients (eight out of 16 with hematological cancer and five out of 13 with solid tumors), with OR = 2.94 (95% CI, 0.48–18.03), p = 0.24 and I^2^ = 0% (**Graph 2**).

### Requirement for ICU Care

**Graph 3:**
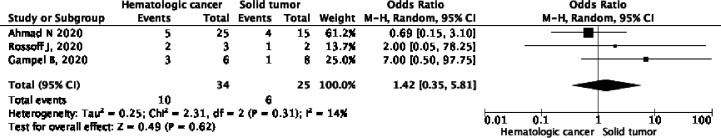
Differences in hospitalization in intensive care for hematological cancer and solid tumors among patients with SARS CoV-2.

Information from three articles [Ahmad et al. (36), Gampel et al. (43), and Rossoff et al. (47)] enabled the evaluation of the need for ICU care in 59 patients (10 out of 34 patients who presented hematological neoplasms *vs.* six out of 25 who presented solid tumors), with OR = 1.42 (CI 95%, 0.35–5.81), p = 0.49, and test for heterogeneity with 1^2^ = 14% (**Graph 3**).

### Requirement for Assistance of Ventilatory Support Maneuvers

**Graph 4:**
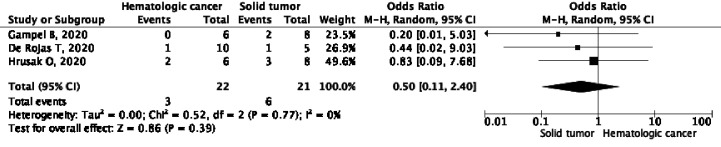
Differences in support with supplemental oxygen for hematological cancer hospitalization and solid tumors among patients with SARS CoV-2.

Tree publications [de Rojas et al. (40), Gampel et al. (43) and Hruzak et al. (45)] described some ventilation assistance maneuver (invasive or non-invasive) in 43 patients (three out of 22 patients with hematological cancer *vs*. six out of 21 with solid tumors), with an OR = 0.50 (CI 95%, 0.11–2.40), p = 0.77, and l^2^ = 0% (**Graph 4**).

## Discussion

In this systematic review and meta-analysis, information was collected from published studies out of which, five were multicenter studies conducted in seven countries (36–44, 47) showing an overall survival of 99.4% in patients with cancer under treatment and having COVID-19. All publications were cross-sectional, reflecting a heterogeneous scenario in the clinical course of these patients. Nonetheless, the objective of this work was not to compare the outcomes of COVID-19 in healthy children or children with other diseases.

Cancer is a priority in global health because it creates a situation of vulnerability with a high risk of serious complications that require prompt attention by specialized personnel (7). In this investigation, the association with COVID-19 showed a mortality of <1% due to the fact that only one death was registered; this value was obtained from the final analysis of the articles. Considering these results, it could be assumed that the current pandemic does not pose a greater risk. This is consistent with a series with similar characteristics in adults. Nonetheless, it should be noted that 14 of the included studies were conducted in cancer hospitals or health centers with conditioned areas for the care of these patients by healthcare professionals specialized in the treatment of complications secondary to chemotherapy. Consequently, it is possible that the same situation in a more general scenario would not yield the same results. Furthermore, it is likely that these results will change once the scientific evidence is updated and data on developing countries are included (such as those located in Latin America and Africa) and with a longer follow-up. For example, in Mexico, the public database of the Ministry of Health showed, up to September 1, 26 deaths in individuals aged <18 years with comorbidity of immunosuppression (total in 367), which includes cancer (50). This information could not be added to the meta-analysis because it is not possible to distinguish cancer from other immunosuppressive conditions in the registry. An additional issue is the variation in the cut-off value of patient age to be considered pediatric. This scenario emerged in the study by Olivia Swann et al. (35), who reported the death of a patient with hematological cancer in a 16–19 age range.

Regarding the comparative analysis between tumor types and the need for hospitalization, no differences were found in the three included studies. This is because in most cases, it coincided with the period immediately after chemotherapy, and the information on the deferral of the sessions is insufficient for analysis (51–53). The same scenario is observed in the need for intensive care in the studies showing the causes, which in addition coincided with febrile neutropenia events. In these studies, the information provided does not allow to differentiate whether it is due to septic shock secondary to bacteremia (all the articles with information on treatment presented empirical antimicrobial regimens) or pneumonia (40).

Analysis on the association between the type of neoplasm and the need for oxygen supply did not show a greater risk between the groups. This is in part because as previously mentioned, most of the cases presented with infections secondary to the myelosuppression derived from the treatment. Up to the date of this review, the clinical presentation compatible with the multisystem inflammatory syndrome in children (MIS-C) (54) has not been reported.

The biases of the included studies are a consequence of the fact that the publications present cross-sectional results and that they limit the possibility of adequately predicting the presence of late outcomes (follow-up bias) as well as the absence of information from the entire *n* of research subjects in the publications, which only describe representative cases, thereby generating selective reporting bias; another bias correspond to the language, since only references in English were included.

It is clear that these results will be subjected to later variations from multicenter groups that currently have cohort designs and from international registries that will include prolonged follow-ups (page of records) adjusted for other potentially confounding variables. Therefore, the authors subscribe to the need to update this review.

With the available information to date, it is not possible to understand the effect that cancer development, age, and type of chemotherapy have on virus infection. In addition, the usefulness of the screening tests in each therapeutic course is unclear, and discriminating the effects of the coronavirus infection from the other complications caused by neoplasia and its treatment represents a challenge.

## Conclusions

In this meta-analysis, no association was found between COVID-19 and the increase in associated mortality in children with cancer. The other outcomes (need for hospitalization/intensive care and ventilation) did not show differences between the types of tumors. This review needs to be updated as soon as information from follow-up studies is available.

## Data Availability Statement

The original contributions presented in the study are included in the article/**Supplementary Material**. Further inquiries can be directed to the corresponding author.

## Author Contributions

ED-A, DÁ-M, and HM-G: manuscript writing. LJ-V and MK-K: substantial contributions and manuscript review. All authors contributed to the article and approved the submitted version.

## Conflict of Interest

The authors declare that the research was conducted in the absence of any commercial or financial relationships that could be construed as a potential conflict of interest.
