# Supplementary material for: Survival and Complications in Pediatric Patients With Cancer and COVID-19: A Meta-Analysis
Source: Front Oncol. 2021 Jan 21;10:608282. doi: 10.3389/fonc.2020.608282 (PMC7861039; doi:10.3389/fonc.2020.608282)
Supplement: Supplementary file 1 [file DataSheet_1.pdf]

**Author, year [reference]**	**Country**	**Design**	**Objective**	**Overall results**	**Outcome:** Global mortality	**Outcome :** Requirement for hospitalization	**Outcome :** Requirement for ICU care	**Outcome :** Requirement for assistance of ventilatory support maneuvers	**Limitations of the study**
**André, 2020** (37)	France	A multicentric descriptive cross-sectional study	To report the diagnosis in pediatric cancer patients and their clinical characteristics	In 19 cancer centers, 33 cases were confirmed by PCR. They focus on five of those who required hospitalization in ICU	0%	N/A	N/A	N/A	They focus only on five out of 33 children who were in the ICU.
**Balduzzi ., 2020** (3)	Italy	Descriptive cross-sectional study	A review by experts and the reporting of positive cases from a hospital facility	PCR diagnosis of five cases: ALL (1 case), osteosarcoma (2 cases), hepatoblastoma (2 cases), rhabdoid tumor (1 case).	0%	N/A	N/A	N/A	This study is included in Hrusak et al.

**Boulad et al., 2020** (38)	United States	Descriptive cross-sectional study	A study that evaluated the diagnosis of COVID-19 in children and caregivers at a New York hospital that assists population with cancer	Out of 335 PCR tests performed on patients and primary caregivers, 20 (11.5%) were confirmed, five of which showed moderate symptoms that required hospitalization	0%	N/A	N/A	N/A	1 patient was hospitalized for non-severe causes, and 3 patients were hospitalized for neutropenia and fever. Even though all of them had a favorable progress, it is not possible to identify what was the preexisting oncological disease in the hospitalized patients.

**De Rojas et al., 2020** (40)	Spain	Descriptive cross-sectional study	To report the clinical characteristics of children with cancer and COVID-19 in Madrid	In 2 months, 1140 PCR tests were performed, out of which 1.3% were positive. There were 15 cases of children, with a median age of 10.6 years, with the following types of cancer: 11 (73%) hematologic neoplasms, 4 (27%) solid tumors, and 4 with transplants	0%	3 out of 10 patients with hematologic neoplasms, and 1 out of the 5 patients with solid tumors required hospitalization.	(N/A)	1 out of the 10 patients with hematologic neoplasms and 1 out of the 5 patients with solid tumors required ventilatory support maneuvers.	(N/A)

**Ferrari., 2020** (41)	Italy	Multicentric descriptive cross-sectional study	To report the characteristics of Italian patients in the region of Lombardy, Italy	Out of 4485 PCR tests, 286 patients were positive, identifying 21 cases of COVID-19 with cancer (number of cases): leukemia (10) sarcomas (5), lymphomas (2), hepatoblastomas (2), central nervous system tumors (1), and colon cancer (1)	0%	N/A	N/A	N/A	Two patients with severe complications were reported. Even though there are no deaths reported it is not possible to know if ICU hospitalization was required.

**Hrusak, 2020** (45)	Italy	Multicentric descriptive cross-sectional study	A brief report of the participation of 32 pediatric hospital centers	There were 200 cancer patients tested with PCR, and eight positive cases were reported. The types of cancer (number of cases) were ALL (2), hepatoblastoma (2), osteosarcoma (1), rhabdoid tumor (1), Ewing's sarcoma (1), Wilms' tumor (1).	0%	N/A)	(N/A)	2 out of 6 patients with hematologic neoplasm and 3 out of 8 patients with solid tumors required ventilatory support maneuvers.	This study includes five patients also reported by Balduzzi et al.
**Zachariah . 2020** (49)	United States	Comparative cross-sectional study	To compare the clinical characteristics of patients infected by COVID-19 who required and did not require hospitalization in the intensive care unit	50 children and teenagers were hospitalized for COVID-19 infection, out of which 2 had hematologic cancer, 2 had solid tumors, and 3 had non-cancer immunosuppres sion	0%	(N/A)	(N/A)	(N/A)	1 patient that ICU required hospitalization was reported. However, it is not possible to identify the clinical characteristics of the imminocompromised patient.

**Garazzino, 2020**<br><br>(44)	Italy	Cross-sectional study	Preliminary report of confirmed cases in Italy	168 confirmed cases were obtained, out of which 110 required hospitalization. 4 out of those 10 had cancer as a comorbidity	0%	(N/A)	(N/A)	(N/A)	It was not possible to know the underlying oncological disease in the 4 patients that were hospitalized with this comorbidity.
**Cesaro 2020**<br><br>(39)	Italy	Cross-sectional study	A total of 334 NFS were performed on 247 patients affected by leukemia or lymphoma and 80 with solid tumors.	The incidence of positive NFS in pediatric patients coming to the hospital for chemotherapy was 3% for all centers, and 3.4% for the northern Italian centers	0%	N/A	N/A	N/A	The individual characteristics of patients are not described. Only characteristics of groups are described.

**Issitt 2020** (46)	UK	Cross-sectional study	Retrospective analysis of children with suspected COVID-19 at a Specialist Children’s Hospital to determine outcomes based on COVID-19 testing status and underlying health vulnerabilities	Between March 1st and May 15th 2020, 166 children (<18 years of age) attended a specialist children’s hospital with clinical features of possible COVID-19 infection. 65 patients (39.2%) tested positive for SARS-CoV-2 virus. 7 patients were undergoing active chemo/radiotherapy and 2 patients had haematologic cancer	0%	N/A	N/A	N/A	Neither the subtypes of cancer nor the outcome variables required for this study were reported in the group of children with this comorbidity.

**Gampel 2020**<br><br>(43)	US	Cross-sectional study	Retrospective evaluation of pediatric hematology, oncology, and HCT patients who were tested for COVID-19 infection from March 10 to April 6, 2020	A total of 174 patients underwent laboratory screening for COVID-19. 19 patients tested positive. Three patients had non-malignant hematologic diagnoses; 14 patients had cancer; and 2 patients had post- allogeneic hematopoietic cell transplantation.	0%	5 out of the 6 patients with hematologic neoplasms, and 4 out of the 8 patients with solid tumors required hospitalization.	3 out of the 6 patients with hematologic neoplasms, and 1 out of the 8 patients with solid tumors required hospitalization in the ICU.	None of the 6 patients with hematologic neoplasm, nor 2 out of the 8 patients with solid tumors required ventilatory support maneuvers.	N/A
**Rossoff 2020**<br><br>(47)	U.S.	Cross-sectional study	To report the experience from an institution with six pediatric oncology patients who tested positive for SARS-CoV-2 infection.	Out of the 6 patients, 1 presented HSCT. Out of the remaining 5, 3 had hematologic neoplasms; and 2 had solid tumors.	0%	All patients with hematologic neoplasmsand solid tumors were hospitalized.	2/3 patients with hematologic neoplasms required ICU hospitalization, as opposed to 1/2 patients with solid tumors.	Not included in the analysis since the only patient who required it was a transplant patient.	Coexistence of neutropenia and fever complications with COVID-19 diagnosis. It is not possible to distinguishthe need for hospitalization for these two causes.
**Flores 2020**<br><br>(42)	México	Cross-sectional study	To report 3 cases from an institution of patients with hematologic disorders, as well as the hematology and HSCT services.	2 out of the 3 patients had ALL and favorable progress. The patient from the HSCT service died.	0%	N/A	N/A	N/A	The series of cases is very small. Therefore, it does not allow to perform comparisons.

**Stokes 2020** (48)	U.S.	Descriptive cross-sectional study	To report two pediatric patients with underlying malignancies.	Both cancer patients had severe disease requiring intensive care.	0%	N/A	N/A	N/A	Since it only includes two patients, it is not possible to perform comparisons with statistical strength.
**Ahmad 2020** (36)	Saudi Arabia	Descriptive cross-sectional study	Report of all outpatient activity and new patients admitted to the pediatric oncology and HSCT services infected by COVID-19 from December 2019 to May 2020,	Out of the 40 patients from the oncology service, 25 had hematologic neoplasms and 15 had solid tumors. One death was recorded in a patient with NHL.	2.5%	N/A	Out of the 25 patients with hematologic neoplasms, 5 required ICU hospitalization. Out of the 15 patients with solid tumors, 4 required ICU hospitalization.	N/A	N/A

ALL: acute lymphoblastic leukemia; ICU: intensive care unit; PCR: polymerase chain reaction; HSCT: hematopoietic stem cell transplant; NHL: Non-Hodgkin lymphoma; NFS: nasopharyngeal swab.

N/A: Not Available
